# A Bayesian network-based approach for identifying risk factors and predicting ischemic stroke in infective endocarditis patients

**DOI:** 10.3389/fcvm.2023.1294229

**Published:** 2024-01-05

**Authors:** Boyi Yuan, Chaobin Wang, Zexin Fan, Chun Liu, Libo Fang, Lin Ma, Wenlong Zou, Guobin Yuan, Guangzhi Liu

**Affiliations:** ^1^ Department of Neurology, Beijing Anzhen Hospital, Capital Medical University, Beijing, China; ^2^ Department of Neurology, Beijing Fangshan District Liangxiang Hospital, Beijing, China; ^3^ Department of Neurology, Mechinka Hospital, Dnipro State Medical University, Dnipro, Ukraine; ^4^ Department of Neurology, Fuxing Hospital, Capital Medical University, Beijing, China

**Keywords:** Bayesian network, infective endocarditis, stroke, risk factor, prediction model

## Abstract

**Objective:**

This study aimed to seek the risk factors and develop a predictive model for ischemic stroke (IS) in patients with infective endocarditis (IE) utilizing a Bayesian network (BN) approach.

**Methods:**

Data were obtained from the electronic medical records of all adult patients at three hospitals between 1 January 2018, and 31 December 2022. Two predictive models, logistic regression and BN, were used. Patients were randomly assigned to the training and test sets in a 7:3 ratio. We established a BN model with the training dataset and validated it with the testing dataset. The Bayesian network model was built by using the Tabu search algorithm. The areas under the receiver operating characteristic curve (AUCs), calibration curve, and decision curve were used to evaluate the prediction performance between the BN and logistic models.

**Results:**

A total of 542 patients [mean (SD) age, 49.6 (15.3) years; 137 (25.3%) female] were enrolled, including 151 (27.9%) with IS and 391 (72.1%) without IS. Hyperlipidemia, hypertension, age, vegetation size (>10 mm), *S. aureus* infection, and early prosthetic valve IE were closely correlated with IS. The BN models outperformed the logistic regression in training and testing sets, with accuracies of 76.06% and 74.1%, AUC of 0.744 and 0.703, sensitivities of 25.93% and 20.93%, and specificities of 96.27% and 90.24%, respectively.

**Conclusion:**

The BN model is more efficient than the logistic regression model. Therefore, BN models may be suitable for the early diagnosis and prevention of IS in IE patients.

## 1 Introduction

Infective endocarditis (IE) is an uncommon, life-threatening disease caused by endocardial surface infections. Neurological complications in IE patients include ischemic/hemorrhagic stroke, mycotic aneurysms, brain abscesses and meningitis, spondylodiscitis, spinal cord abscesses, and encephalopathy (1). Among these, ischemic stroke (IS) is one of the most disastrous complications of IE, accounting for 16%–25% of patients (2–4), and ultimately leading to high mortality and morbidity (5, 6). Hence, the accurate and timely identification of IS in IE patients is crucial, as it could improve disease prognosis and quality of life. The risk factors for ischemic and hemorrhagic stroke include male sex, older age, prior IE or stroke, *S. aureus* infection, fungal infection, atrial fibrillation, multiple vegetation, mitral valve vegetation, valvular abscess, large vegetation (>10 mm), and rheumatic heart disease (4, 7–10). Although predictive models (e.g., ER French Calculator) have been proposed to assess the risk of embolic events in IE patients, they lack external validation (11, 12). Given the unavailability of universally accepted methods for predicting future embolic events, particularly IS, in IE patients, highly predictive models are urgently needed for early IS identification and diagnosis. Herein, we built models separately for IS in IE patients using the Bayesian network (BN) method and logistic regression. We compared the performance of the models using areas under the curve (AUCs), calibration curve, and decision curve analysis (DCA).

## 2 Material and methods

### 2.1 Setting and data sources

Data were obtained from the electronic medical records of all adult patients at three hospitals between 1 January 2018, and 31 December 2022. A randomization method was used for patient's allocation. Data were analyzed from 1 February to 31 September 2023. The inclusion criteria were: (i) age ≥ 18 years; (ii) diagnosis of possible and definite IE in accordance with the modified Duke criteria (13). The exclusion criteria were: (i) right-sided IE cases were excluded unless a concomitant left-sided infection was present, (ii) patients with other tumors or gravidity (14), and (iii) patients with missing clinical data such as demographic information, medical history, comorbidities, echocardiographic characteristics, or laboratory examination. All patient underwent transesophageal echocardiography (TEE), and most of them (*n* = 425) also underwent transthoracic echocardiography (TTE) examination. M-mode was used to measure left ventricular ejection fraction [LVEF], left atrial diameter [LAD], left ventricular end-diastolic diameter [LVEDD]. High-mobility refers to highly mobile phenomena of vegetation mass on transesophageal echocardiography (TEE) (15, 16), but so far there are no literatures dealing with the threshold value of the width of mobility. To ensure the standardization of variable across operators, we selected 10 mm as the threshold, since one previous study revealed vegetation size >10 mm were more frequently seen in IE patients complicated by embolic events (17). Early prosthetic valve IE refers to IE that occurs within 1 year after implantation of a prosthetic valve. All participating hospitals' ethics and research committees approved the study.

### 2.2 Outcome variables

To diagnose the target outcome, IS, medical histories, clinical examinations, magnetic resonance angiography scan, and cranial magnetic resonance imaging results were acquired prior to cardiac surgery. Two independent, blinded neurologists confirmed the diagnosis of IS according to medical history, clinical examination and results of cerebral magnetic resonance imaging (MRI) and magnetic resonance angiography scans.

### 2.3 Data extraction and quality control

Two researchers extracted the data. Input data, including demographic information (sex, age), medical history [hypertension, hyperlipidemia, diabetes, and atrial fibrillation (AF)], comorbidities, echocardiography (affected valve, vegetation size, vegetation mobility, LVEF, LAD, LVEDD, and regurgitation), electrocardiogram, and laboratory tests (blood culture results, high sensitivity troponin I (Hs-TNI), estimated glomerular filtration rate [eGFR], hemoglobin, and D-dimer, were gained from the hospital's electronic medical record system upon completion of initial admission. The data collection process was standardized, and the researchers were already familiar with this process before initiating data retrieval. A double-entry approach was used. Medical records were reviewed, and discrepancies were corrected during the review period.

### 2.4 Data processing for potential variables

To establish the predictive model, we consulted relevant literature and processed the data accordingly based on previous studies. In two meta-analysis studies, larger vegetations (>10 mm) significantly increase the rates of embolism and mortality in patients (9), An infection with *S. aureus* is linked to a higher risk of short-term embolic events (EEs) (18). EEs are common among patients with IE and are associated with mitral valve endocarditis, as well as the usage of antiplatelet drugs or intravenous drug (19). Moreover, a remarkable association was identified between elevated plasma D-dimer levels and the risk of IS (20). Thoker et al, conducted a study which indicated that patients with IE and elevated levels of cardiac troponin I were associated with an increased odds of adverse clinical outcomes, including cerebral mycotic aneurysm and meningitis (21). Biostatistical literature suggests that if the rate of missing data in a dataset exceeds 30%, the data will lose its measure of confidence. Most of these attributes are missing due to test failures and time conflicts. Hence, in our study, instances with more than 9 missing attributes (out of 27) were excluded from the dataset. Finally, combined with traditional IS risk factors, we identified 27 variables, including age, hypertension, hyperlipidemia, diabetes, and AF as the dataset. We first screened for potential IS-related risk factors to build a predictive model using logistic regression. The data were splited into training and testing sets in a 7:3 ratio using a random number table. IS was then predicted using BN and logistic regression models. A Tabu search algorithm was utilized to establish the BN model. Model performance was evaluated by use of test sets. The IS-related factors were measured and coded before building the BN model.

When constructing predictive models for dichotomous outcomes, it is recommended to have a sample size that is at least 10 times larger than the number of independent variables. Due to the inclusion of 11 variables in the multivariate analysis, a minimum sample size of 110 per group was required. Eventually, we included 151 and 391 IE patients with and without IS, respectively. Therefore, our sample size was sufficient.

### 2.5 BNs

The BN model is represented as a directed acyclic graph (22). The nodes in the graph represent random variables, while the directed edges symbolize the probabilistic dependencies between variables in the model. If a directed arc exists from *X*_1_ to *X*_2_, it represents *X*_1_ leading to *X*_2_; *X*_1_ is the parent node, and *X*_2_ is the child node. The state of each node's parent node is represented by a conditional probability distribution table associated with that node. The BN represents the joint probability distributions of random variables $X=\{X_{1},\ldots ,X_{n}\}$; thus, a probability expression can be obtained.

$$P(X_{1},\ldots ,X_{n})=P(X_{1})P(X_{2}|X_{1})\ldots P(X_{n}|X_{1},X_{2},\ldots X_{n-1})=\prod_{1}^{n}P(X_{i}|\pi (X_{i}))$$

where *P* (*X_i_*) represents the collection of the parents of *X_i_*; $\pi (X_{i})\subseteq \{X_{1}\;\ldots ,\;X_{i-1}\}\subseteq \{X_{1}:::,\;X_{i-1}\}$ (23).

For each instance, 27 random variables were derived from the patient data. Given that too many variables add unnecessary complexness to the BN structure, we used univariate analysis to screen the nodes. The Tabu search algorithm was then used to construct the best model.

### 2.6 Statistical analysis

SPSS Statistics for Windows (version 23.0) was utilized for conducting the statistical analyses. Continuous variables were expressed as mean ± standard deviation or median (interquartile range). Categorical variables were presented as number of subjects (*n*) and percentage (%). Categorical variables were analyzed utilizing the *χ*^2^ test. Normally distributed data and non-normally distributed data are analyzed using the Student's *t*-test and the Mann–Whitney *U-*test, respectively. Variables associated with IE-related IS were evaluated using binary logistic regression analysis. In the univariate analysis, variables in association with the outcome at a significance level of <0.05 are deemed potential candidates for further consideration in the subsequent multivariate analysis. Receiver operating characteristic (ROC) curve analysis was utilized to evaluate the predictive models and calculate the AUCs. The Delong test was utilized to evaluate the statistical significance of the disparity among AUC values. To assess the difference between predicted values and true values and plot a calibration curve, the Hosmer–Lemeshow (HL) test was utilized. DCA was performed to access the net benefits of each predictive model. The Tabu Search algorithm was employed to build a BN model. The maximum likelihood estimation method was utilized for parameter estimation of the BN model. R Studio 4.2.2 was used to establish the BN model. To visualize the BN topology, we employed Netica32 software (Norsys Software Corp., Vancouver, Canada).

## 3 Result

### 3.1 Patients selection

**Figure 1:**
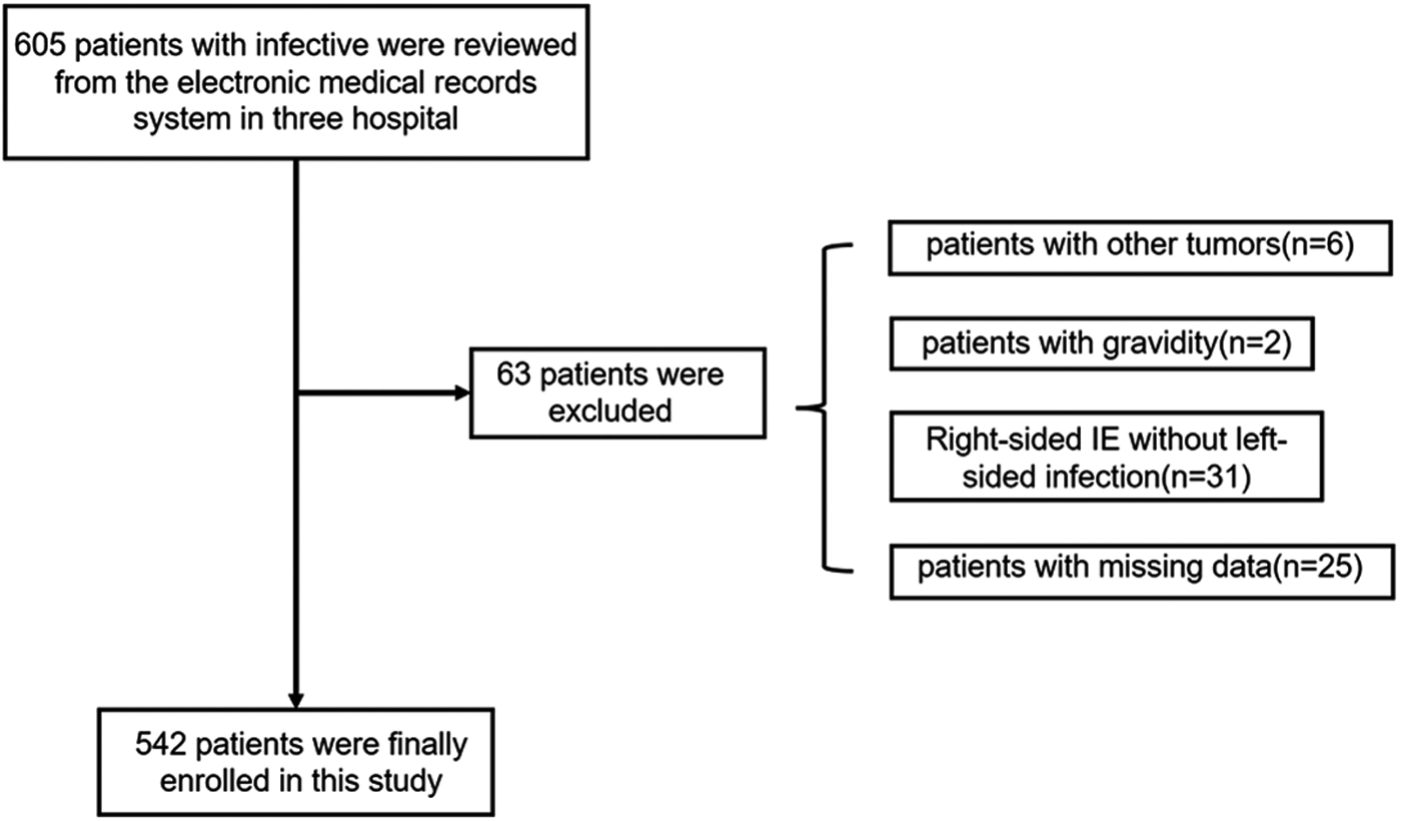
Flowchart describing the screening of patients with infective endocarditis (IE) patients.

Of the 605 patients with IE, 63 individuals were excluded from the study due to right-sided IE, other comorbidities, or missing data. Finally, 542 patients [mean (SD) age, 49.6 (15.3) years; 137 (25.3%) female] were enrolled, including 151 (27.9%) with IS and 391 (72.1%) without IS (Figure 1 and Table 1). IS was confirmed before cardiac surgery (interventions) among all IE patients. In this study, some IE patients were on anticoagulation due to prosthetic valve replacement or AF, but no significant difference in anticoagulation management was found between the patients with IS and those without IS [55 (14.1%, 55/391) vs. 26 (17.2%, 26/151), *P* = 0.43]. Moreover, thirteen patients with cardiac device-associated IE were included in this study. All IE patients underwent blood culture, and the results are presented in Table 2. Although *Viridans group streptococci* was the most common pathogen, no significant difference was found between it and other pathogen isolated (*P *= 0.072).

**Table 1:** Baseline data of patients with infective endocarditis (IE).

Variables	IE without IS (*N* = 391)	IE with IS (*N* = 151)	*P*-value
Sex (Male)	299 (76.5)	106 (70.2)	0.1627
Age, mean (SD), years	47 (34,58)	59 (51,65)	**<0.001**
Current smoking	108 (27.6)	46 (30.5)	0.5813
Current drinking	80 (20.5)	33 (21.9)	0.8102
Hypertension	72 (18.4)	57 (37.7)	**<0.001**
Hyperlipidemia	10 (2.6)	12 (7.9)	**0.009**
Diabetes	53 (13.6)	30 (19.9)	0.09
Atrial fibrillation	20 (5.1)	20 (13.2)	**0.0022**
Cardiac function (class Ⅲ-Ⅳ)	145 (37.1)	52 (34.4)	0.6349
Early prosthetic valve IE	13 (3.3)	15 (9.9)	**0.0037**
Late prosthetic valve IE	32 (8.2)	9 (6.0)	0.4861
Affected valve			
Mitral	222 (56.8)	98 (64.9)	0.1038
Aortic	237 (60.6)	90 (59.6)	0.9062
Vegetation size (>10 mm)	145 (37.1)	74 (49.0)	**0.0148**
Vegetation mobility	202 (51.7)	97 (64.2)	**0.011**
LVEF (%)	61 (57, 66)	61 (57, 66)	0.8057
LAD (mm)	42 (37, 46)	43 (38, 46)	0.201
LVEDD (mm)	57 (52, 62)	56 (51, 61)	0.1394
Mitral regurgitation	235 (60.1)	99 (65.6)	0.2831
Aortic regurgitation	201 (51.4)	79 (52.3)	0.9248
Perivalvular abscess	25 (6.4)	15 (9.9)	0.2187
*S. aureus*	7 (1.8)	9 (6.0)	**0.0193**
Hypohepatia			0.5921
Normal	340 (87.0)	128 (84.8)	
Low	41 (10.5)	19 (12.6)	
Middle	4 (1.0)	3 (2.0)	
High	6 (1.5)	1 (0.7)	
Hemoglobin (g/L)	109 ± 21.4	104 ± 21.5	**0.047**
eGFR (ml/min/1.73 m^2^)	103.5 (85.7, 118.5)	88.9 (58.9, 104.6)	**<0.001**
D-dimer (ng/ml)	339 (207, 674)	481 (323, 891)	**<0.001**

IE, infective endocarditis; AF, atrial fibrillation; eGFR, estimated glomerular filtration rate; IS, ischemic stroke; LVEDD, left ventricular end-diastolic diameter; LVEF, left ventricular ejection fraction; LAD, left atrium diameter; *S. aureus*, *staphylococcus aureus*.

*P*-values less than 0.05 have been highlighted in bold.

**Table 2:** Microbiological etiology of infective endocarditis.

Causal agent	IE With IS	IE Without IS
*S. aureus*	9	7
*Coag Neg staph*	10	18
*Viridans group streptococci*	22	55
*Enterococci*	5	8
*HACEK*	3	0
other	6	13
Culture negative	99	287

*S. aureus*, *Staphylococcus aureus*; Coag Neg staph, coagulase-negative staphylococci; HACEK, Haemophilus spp., Aggregatibacter (formerly Actinobacillus) actinomycetemcomitans, Cardiobacterium hominis, Eikenella corrodens, and Kingella species.

### 3.2 Risk factors for IS

The risk factors associated with IS were hypertension [odds ratio (OR), 2.687; 95% confidence interval (CI), 1.771–4.076; *P* < 0.001], older age (OR, 1.049; 95% CI, 1.034–1.064; *P* < 0.001), hyperlipidemia (OR, 3.289; 95% CI, 1.390–7.784; *P *= 0.007), atrial fibrillation (AF) (OR, 2.832; 95% CI, 1.477–5.430; *P *= 0.002), early prosthetic valve IE (OR, 3.207; 95% CI, 1.488–6.913; *P *= 0.003), vegetation size (>10 mm) (OR, 1.630; 95% CI, 1.116–2.383; *P* = 0.012), vegetation mobility (OR, 1.681; 95% CI, 1.141–2.476; *P *= 0.009), *S. aureus* infection (OR, 3.477; 95% CI, 1.271–9.511; *P *= 0.015), estimated glomerular filtration rate (eGFR) (OR, 0.983; 95% CI, 0.977–0.989; *P *< 0.001), hemoglobin (OR, 0.990; 95% CI, 0.982–0.999; *P *= 0.035), D-dimer (OR, 1.000; 95% CI, 1.000–1.000; *P* = 0.262).

In the logistic regression models, we incorporated four variables that exhibited statistical significance, including older age (OR, 1.037; 95% CI, 1.019–1.055; *P* < 0.001), vegetation mobility (OR, 1.642; 95% CI, 1.042–2.590; *P* = 0.017), *S. aureus* infection (OR, 4.5; 95% CI, 1.375–14.733; *P* = 0.013), and early prosthetic valve IE (OR, 3.529; 95% CI, 1.544–8.066; *P* = 0.003) (Table 3).

**Table 3:** Risk factors of iS in infective endocarditis (IE) patients: multivariate binary logistic regression analysis.

Category	B	SE	Wald	OR (95% CI)	*P-*value
Age	0.036	0.009	16.764	1.037 (1.019, 1.055)	**<0.001**
Hypertension	0.478	0.245	3.804	1.613 (0.998, 2.607)	0.051
Hyperlipidemia	0.461	0.501	0.849	1.586 (0.595, 4.232)	0.357
D-dimer	0.000	0.000	0.123	1.000 (1.000, 1.000)	0.725
Early prosthetic valve IE	1.261	0.422	8.939	3.529 (1.544, 8.066)	**0.003**
*S. aureus*	1.504	0.605	6.179	4.5 (1.375, 14.733)	**0.013**
Vegetation size (>10 mm)	0.383	0.232	2.731	1.466 (0.931,2.309)	0.098
Vegetation mobility	0.496	0.232	4.558	1.642 (1.042, 2.590)	**0.033**
AF	0.283	0.376	0.569	1.328 (0.636, 2.772)	0.451
eGFR	−0.005	0.004	1.932	0.995 (0.987,1.002)	0.165
Hemoglobin	−0.001	0.005	0.049	0.999 (0.989,1.009)	0.825

IE, infective endocarditis; IS, ischemic stroke; AF, atrial fibrillation; eGFR, estimated glomerular filtration rate; *S. aureus*, *Staphylococcus aureus*.

*P*-values less than 0.05 have been highlighted in bold.

### 3.3 BN structure

**Figure 2:**
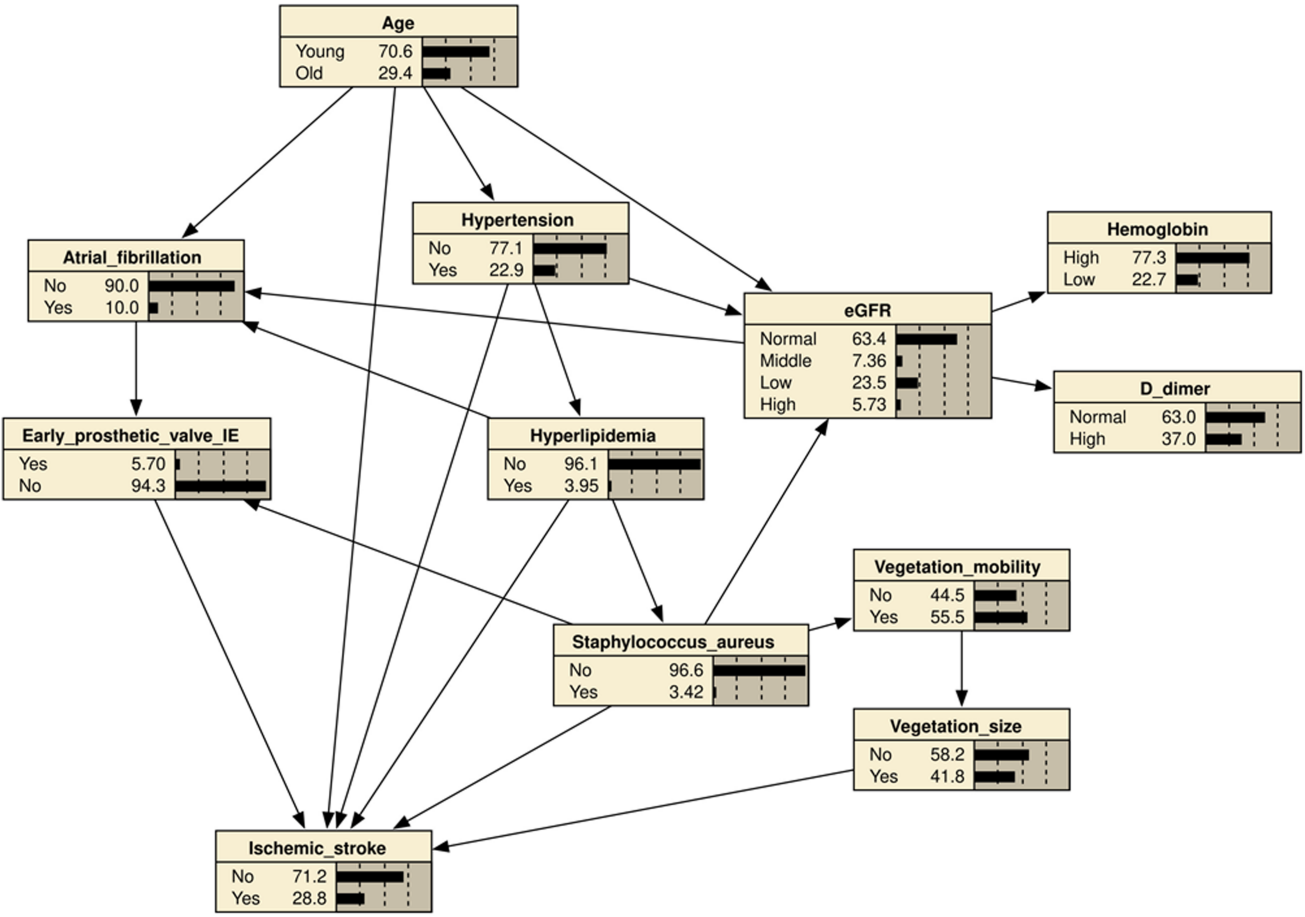
Bayesian network (BN) for predicting the occurrence of ischemic stroke (IS) in patients with infective endocarditis (IE). The BN model used 11 variables selected by univariate logistic regression analysis. Hypertension, age, hyperlipidemia, atrial fibrillation, early prosthetic valve IE, Vegetation size (≥10 mm), vegetation mobility, *S. aureus* infection, eGFR, Hemoglobin, D-dimer.

It was based on a BN model consisting of 21 directed edges and 12 nodes. The nodes represented IS, eGFR, hyperlipidemia, hypertension, age, AF, early prosthetic valve IE, vegetation mobility, vegetation size (>10 mm), *S. aureus* infection, D-dimer, and hemoglobin. The nodes directly linked to IS included age, hyperlipidemia, hypertension, vegetation size (>10 mm), early prosthetic valve IE, and *S. aureus* infection. AF was indirectly associated with IS through its association with early prosthetic valve IE. Vegetation mobility was indirectly associated with IS through its correlation with vegetation size (>10 mm). The eGFR was indirectly associated with IS through its link with AF and early prosthetic valve IE (Figure 2). Based on maximum likelihood estimation, the conditional probabilities of each node in the network (Table 4) were estimated. The variables predictive of IS were hyperlipidemia, age, hypertension, early prosthetic valve IE, vegetation size (>10 mm), and *S. aureus* infection.

**Table 4:** The conditional probability table of the training set based on ischemic stroke (IS) as the target node.

Age ≥60	Vegetation size (>10 mm)	Hyperlipidemia	*S. aureus*	Early prosthetic valve IE	Hypertension	IE with IS	IE without IS
Yes	No	No	No	No	No	0.34	0.66
Yes	No	No	No	No	Yes	0.35	0.65
Yes	No	No	Yes	No	No	1	0
Yes	No	No	Yes	No	Yes	1	0
Yes	Yes	No	No	No	No	0.45	0.55
Yes	Yes	No	No	No	Yes	0.6	0.4
Yes	Yes	No	Yes	No	No	1	0
Yes	Yes	No	Yes	No	Yes	1	0
Yes	No	No	No	Yes	No	1	0
Yes	No	No	No	Yes	No	1	0
Yes	Yes	No	No	Yes	No	1	0
No	No	No	No	No	No	0.09	0.91
No	No	No	No	No	Yes	0.3	0.7
No	No	No	Yes	No	No	0.4	0.6
No	Yes	No	No	No	No	0.24	0.76
No	Yes	No	No	No	Yes	0.3	0.7
No	No	No	No	Yes	No	0.29	0.71
No	Yes	No	Yes	No	No	1	0
No	No	No	No	Yes	Yes	0.66	0.33
No	Yes	No	No	Yes	No	0.5	0.5
No	Yes	No	No	Yes	Yes	1	0
Yes	No	Yes	No	No	Yes	0.66	0.33
Yes	Yes	Yes	No	No	Yes	0.5	0.5
No	No	No	No	Yes	Yes	0.67	0.33
Yes	Yes	No	No	Yes	No	1	0
No	Yes	No	No	Yes	No	0.5	0.5
Yes	No	No	No	Yes	Yes	1	0
No	No	Yes	No	No	Yes	0.5	0.5
No	No	Yes	No	No	No	1	0
No	Yes	Yes	No	No	No	0	1
No	Yes	Yes	No	No	Yes	0.5	0.5

IE, infective endocarditis; IS, ischemic stroke; *S. aureus*, *staphylococcus aureus*.

### 3.4 Model performance evaluation

**Figure 3:**
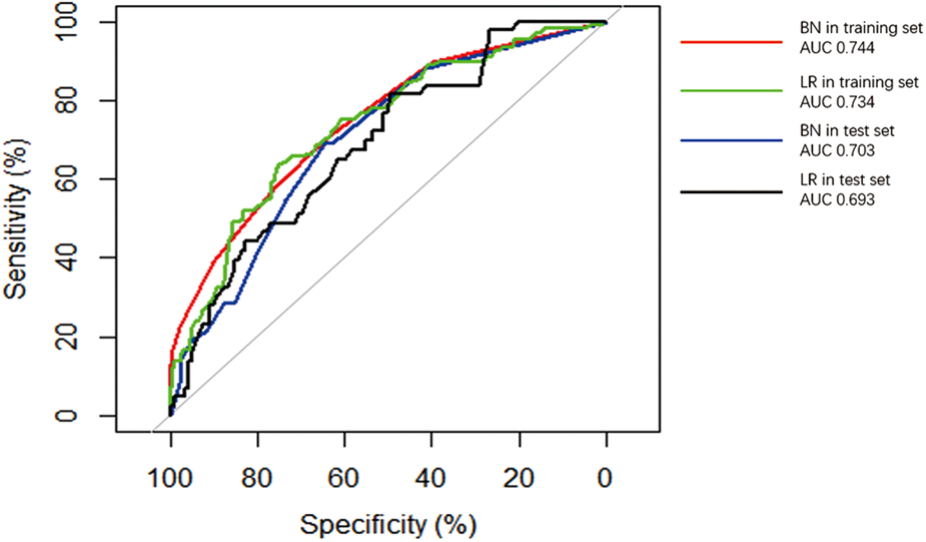
Receiver operating characteristic (ROC) curves of Bayesian network (BN) model and logistic regression (LR) model for predicting ischemic stroke (IS) in patients with infective endocarditis (IE). The areas under the curve (AUCs) of the BN model predicting IS was 0.744 and 0.703 in (red line) training and (blue line) test datasets, respectively. The AUC of the logistic regression LR model predicting IS was 0.734 and 0.693 in the (green line) training and (black line) test datasets.

**Figure 4:**
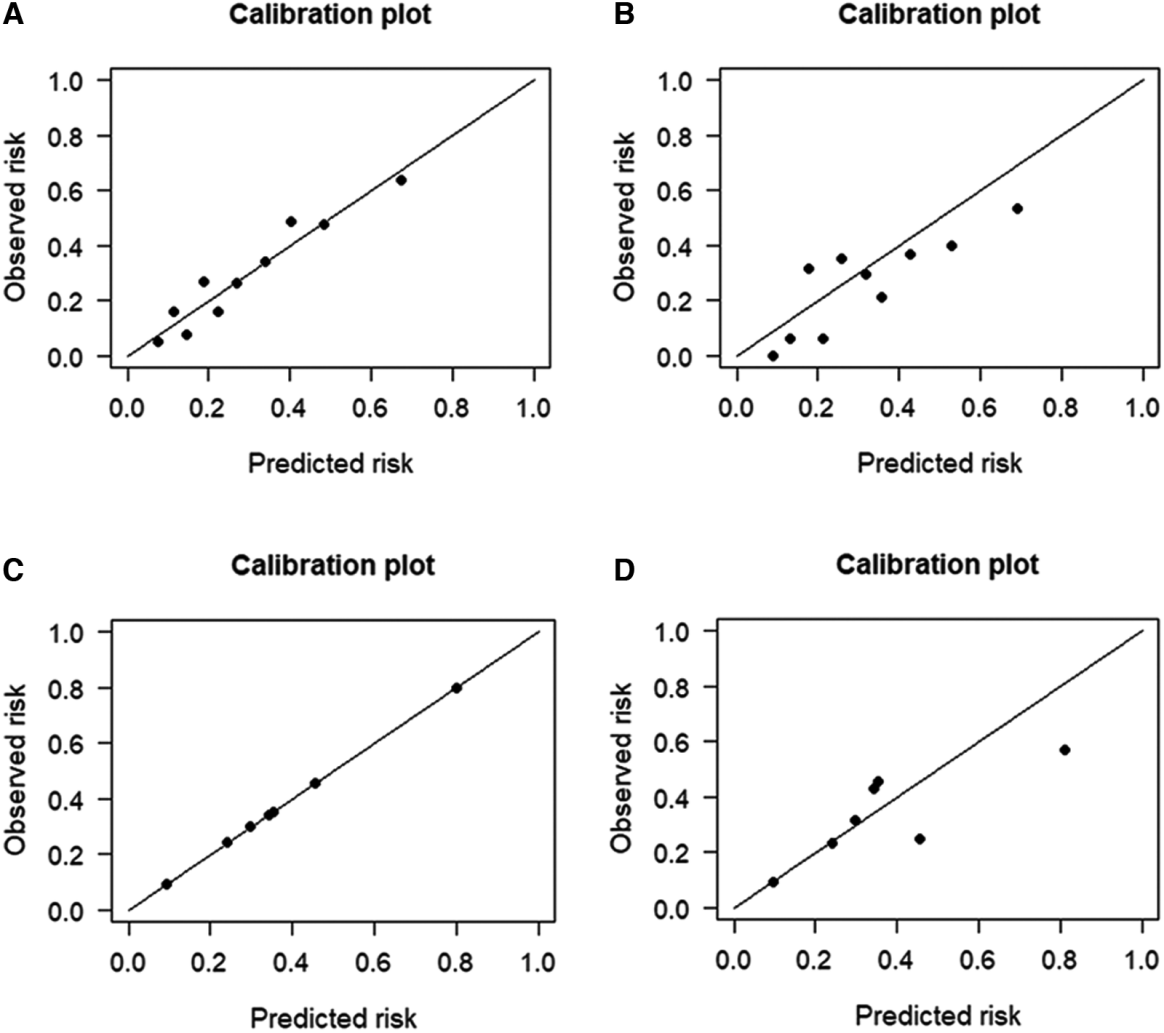
Calibration plots for the four prediction models in both cohorts. The calibration plots showed that the predicted risk of ischemic stroke (IS) agreed well with the observed risk, in either the logistic regression model of (**A**) training and (**B**) testing datasets, or in the Bayesian network model of (**C**) training and (**D**) testing datasets.

**Figure 5:**
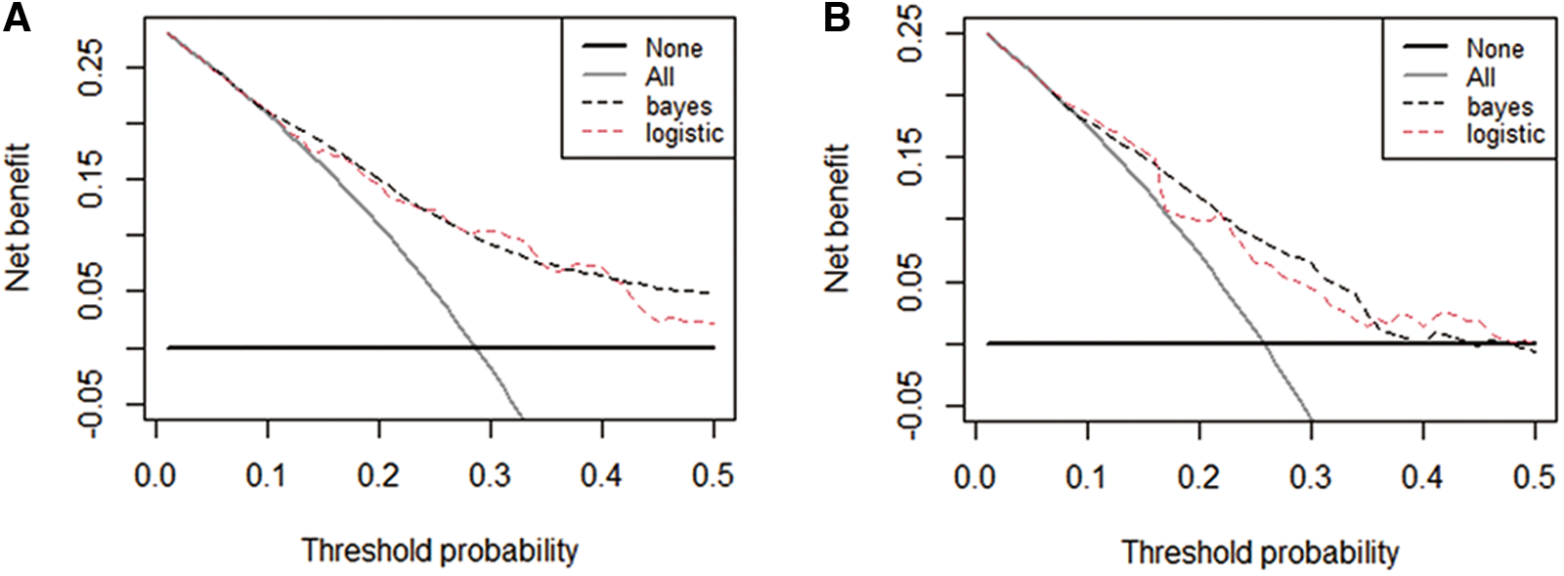
Decision curve analysis (DCA) curves plots for the four prediction models in both cohorts. DCA curves of each model for predicting the probability of IS in the training set (**A**) and test set (**B**) The horizontal and vertical axes represent the threshold probability and net benefit, respectively. The lines between the horizontal axis and vertical axis and vertical axis display the benefits of the different predictive variables. Compared to the logistic regression model, The decision curve showed that the Bayesian model for IS performed equally well in the training and test sets.

The performance of the two models was assessed by examining metrics including accuracy, AUC, specificity, sensitivity, calibration curve, and decision curve (Table 5). The logistic regression predictive model exhibited accuracies of 73.4% and 74.1%, AUCs of 0.734 and 0.693, sensitivities of 25.9% and 30.23%, and specificities of 92.5% and 89.43% in the training and testing datasets, respectively. On the other hand, The BN model exhibited accuracies of 76.06% and 74.1%, AUCs of 0.744 and 0.703, sensitivities of 25.93% and 20.93%, and specificities of 96.27% and 90.24%. No statistically significant difference in the AUC values was shown between the logistic regression and BN models (Figure 3). The Delong test was performed in the training and testing cohorts, respectively, with *P-*values >0.05 (*P* = 0.8102 and *P* = 0.849). Moreover, the calibration curves demonstrated that the BN model outperformed the logistic model regarding the degree of fit between the actual and predicted probabilities (Figure 4). Both the logistic regression model and BN model demonstrated good calibration in either training sets [BN: *P* = 0.999, *χ*^2^ < 0.001, degrees of freedom (df) = 8; logistic regression: *P* = 0.6256, *χ*^2^ = 6.193, df = 8]] or testing sets (BN: *P* = 0.3496, *χ*^2^ = 8.914, df = 8; logistic regression: *P* = 0.1469, *χ*^2^ = 12.097, df = 8) as indicated by the Hosmer–Lemeshow test. DCA showed a non-significant difference in net benefits between the BN and logistic regression models for predicting IS in the training or test sets (Figure 5).

**Table 5:** The performance of different predictive models.

Model	Accuracy	AUC	Sensitivity	Specificity
Bayesian network (training set)	76.06%	0.744	25.93%	96.27%
Logistic regression (training set)	73.4%	0.734	25.9%	92.5%
Bayesian network (test set)	74.1%	0.703	20.93%	90.24%
Logistic regression (test set)	74.1%	0.693	30.23%	89.43%

## 4 Discussion

In the present study, we employed a BN-based approach with the available data to predict the occurrence of IS. Hyperlipidemia, *S. aureus* infection, early prosthetic valve hypertension, age, and vegetation size (>10 mm) were directly correlated with IS, whereas AF, eGFR, and vegetation mobility were indirectly related with IS. On the other hand, the BN model achieved superior or equivalent predictive performance for IS to the logistic regression model.

Consistent with previous studies (4, 16, 18), our study demonstrated that *S. aureus* infection was associated with an increased risk of IS. The mechanisms underlying IS remain largely unknown. Hence, we speculate that infections may cause inflammation and procoagulation, including vascular leakage, endothelial injury, and hypotension. These events lead to atherosclerotic plaque instability and rupture and arterial occlusion, ultimately result in arterial thrombosis (24–26). Further studies are required to address this issue.

The risk of stroke is remarkably increased in multiple vegetation types, including mitral valve vegetation, valvular abscess, and large vegetation (>10 mm) (4, 7, 9, 27). Our study found that larger vegetation was significantly associated with IS. Additionally, vegetation mobility was indirectly related to IS through its association with vegetation size (>10 mm). Concurrently, two distinct studies demonstrated that vegetation size > 10 mm and high vegetation mobility were predictors of embolic events (28–31). Therefore, to prevent IS, an echocardiographic examination of the cardiac structure and early management should be routinely performed in IE patients.

We observed a significant association between early prosthetic valve IE and IS. This association is noteworthy because prosthetic valve endocarditis (PVE) accounts for approximately 30% of IE cases worldwide, with in-hospital mortality of 20%–40%, partially due to cerebrovascular events or stroke (32–34). *S. aureus* is the most prevalent pathogen implicated in PVE, and patients with *S. aureus* PVE have notably higher rates of mortality in hospital settings (33, 35). Hence, accurate and dynamic monitoring of *S. aureus*-related PVE and timely intervention could improve clinical outcomes.

In our study, hypertension, older age, and hyperlipidemia were directly associated with IS, implying that such risk factors require strict monitoring and management in IE patients, especially in older adults, to prevent IS. In a cohort of 507 patients diagnosed with native left-sided IE, approximately 10.3% experienced new-onset atrial AF, an established risk factor for IS. Notably, patients with pre-existing or newly developed AF demonstrated significantly higher 1-year in-hospital mortality rates compared to patients without AF (36). The impact of IE on heart valves can lead to subsequent heart failure and valvular insufficiency, which in turn can contribute to the onset or progression of AF (37). Our results revealed that AF was indirectly linked to IS through its association with early prosthetic valve IE utilizing the BN model, further provingour viewpoint. Nonetheless, more evidence is needed to address this issue.

BN models offer several advantages in the medical field. Bayesian networks can handle missing data, a feature distinct from logistic models, in which missing values are not allowed between covariates. Because missing data is very common in clinical practice, BN is well-suited for establishing diagnostic models. Moreover, BN typically models structures in a domain, and the results are intuitively visible. BN enables quantitative risk assessment of selected clinically relevant outcomes. As shown in Table 4, an elderly patient without hypertension, *S. aureus* infection, vegetation size (>10 mm), vegetation mobility, hyperlipidemia and early prosthetic heart valve had a probability of 0.34 for concurrent IS; In the presence of vegetation size (>10 mm), the probability increased to 0.45. The probability further rose to 0.6 if these patients also had hypertension. Thus, our results can be easily applied in clinical practice and is very beneficial for early IS detection and diagnosis of patients. Furthermore, these factors may contribute to the prevention of initial and recurrent IS.

This study's calibration results revealed an agreement between the BN and logistic regression models. The DCA curves indicated that BN and the logistic regression model had good net benefits in predicting IS. Additionally, the performance of the BN model was better or no worse than the traditional logistic regression model, in the context of accuracy, AUC, specificity and sensitivity. More importantly, the logistic model assumes that each variable is independent and ignores the relationship between risk factors, while the BN model builds a network model through the deep mining of data, which further reveals the interaction between variables and more realistically reflects the effect of each risk factor on IS. However, in clinical trials, missing data for the predictive model can potentially render the prediction unable to proceed. On the contrary, the BN is built upon disease-related knowledge, effectively harnessing the available data to uncover valuable information and unveil the intricate interactions among multiple factors.

To our knowledge, this study, for the first time, construct a BN model for predicting IS in IE patients, and it was more efficient than the logistic regression model. Age, early prosthetic valve IE, vegetation size (>10 mm), hypertension, hyperlipidemia, and *S. aureus* infection were significant predictors of IS in our Chinese cohort. Further research is needed to investigate the risk factors for IS in patients with IE and to determine their causal relationships. This research aims to enhance strategies for disease prevention and eventually reduce the embolic rate and mortality among IE patients.

## 5 Limitations of the study

This study had some limitations. The first limitation in our study is the small sample size, primarily due to the rarity of IE. Second, the BN-directed edges describe the probabilistic dependencies between variables, instead of a causal relationship. Third, the study is exposed to a detection bias for the determination of the baseline characteristics, since the samples were assembled retrospectively. Fourth, considering the predictors at admission in this study, the model may not study the predictive value of their modifications after the initial evaluation. Last but not least, we didn't handle mortality events and included a composite outcome of stroke and mortality, as this is a cross-sectional study to screen the risk factors and develop a predictive model for IS. Therefore, future large-scale prospective studies are needed.

## Data Availability

The raw data supporting the conclusions of this article will be made available by the authors, without undue reservation.
